# Isolated testicular immunoglobulin G4-related disease mimicking malignancy: a rare case report

**DOI:** 10.1097/MS9.0000000000005592

**Published:** 2026-08-26

**Authors:** Sushant Shah, Bishal Panthi, Siddhartha Karn, Abhaya Acharya

**Affiliations:** a Maharajgunj Medical Campus, Institute of Medicine, Tribhuvan University, Kathmandu, Nepal; b Department of Pathology, Tribhuwan University Teaching Hospital, Kathmandu, Nepal

**Keywords:** autoimmune pseudotumor, immunoglobulin G4-related disease, orchiectomy, testicular mass

## Abstract

**Background:**

Immunoglobulin G4-related disease (IgG4-RD) is a systemic fibroinflammatory disorder that can involve virtually any organ; however, isolated testicular disease remains exceptionally rare. Its ability to mimic malignancy, both clinically and radiologically, frequently results in an unwarranted orchiectomy.

**Case presentation:**

We report a 49-year-old man from eastern Nepal who presented with a 3-day history of painless right testicular swelling. Examination revealed a firm, non-tender mass with normal serum tumor markers (beta-human chorionic gonadotropin, alpha-fetoprotein, and lactate dehydrogenase). Scrotal ultrasonography demonstrated multiple hypoechoic lesions without internal vascularity, raising suspicion for malignancy. A right high inguinal orchiectomy was performed. Histopathological evaluation revealed dense lymphoplasmacytic infiltration, storiform fibrosis, and a predominance of IgG4-positive plasma cells (40–45%), confirming IgG4-related disease of the testis. The postoperative course was uneventful.

**Discussion:**

This case exemplifies the diagnostic challenge posed by isolated testicular IgG4-RD, a benign yet deceptive entity that can be misinterpreted as testicular cancer. Despite characteristic radiological features and normal tumor markers, clinical suspicion for malignancy often drives unnecessary surgery. Recognition of IgG4-RD in the differential diagnosis is essential, as timely medical therapy with corticosteroids can achieve remission and preserve the testis.

**Conclusion:**

Isolated testicular IgG4-RD should be considered in patients presenting with painless testicular enlargement and normal tumor markers. Early identification and appropriate immunopathological evaluation may help avoid unnecessary orchiectomy and facilitate individualized management, including surveillance or immunosuppressive therapy, depending on disease extent and clinical activity.

## Introduction

Immunoglobulin G4-related disease (IgG4-RD) is a recently recognized systemic inflammatory condition that affects multiple organs^[^1^]^. The disease is characterized by mass-forming lesions with specific microscopic features, including dense collections of lymphocytes and plasma cells (particularly IgG4-positive plasma cells), distinctive scar-like tissue formation (storiform fibrosis), and inflammation of small blood vessels^[^2^]^. Patients with this condition typically have elevated serum IgG4 levels^[^3^]^.

IgG4-RD was first identified in patients with autoimmune pancreatitis but is now known to affect many organ systems throughout the body^[^4^]^. These include the salivary glands, tissues around the eyes, bile ducts, lymph nodes, kidneys, aorta, soft tissues in the abdomen, thyroid gland, lungs, brain coverings, breast, prostate, and skin^[^5^]^. The genitourinary system, which includes the kidneys, ureters, bladder, urethra, prostate, testes, and penis, can also be involved^[^6^]^. However, involvement of the male reproductive organs remains uncommon, especially when it occurs alone without other organ involvement.

HIGHLIGHTS

- Isolated testicular involvement in immunoglobulin G4-related disease (IgG4-RD) is extremely rare.
- The lesion clinically and radiologically mimicked a testicular malignancy.
- Histopathology and IgG4 immunostaining confirmed the diagnosis.
- Awareness of this entity can help avoid unnecessary orchiectomy.
- This highlights the importance of including IgG4-RD in the differential diagnosis of testicular masses.

Testicular involvement in IgG4-RD is extremely rare and creates significant diagnostic difficulties. Previous case reports have shown that these testicular lesions can closely resemble cancer both on physical examination and on imaging studies^[^7^]^.

This similarity often leads surgeons to perform radical orchiectomy (complete removal of the testicle), believing they are treating cancer^[^8^]^. This situation is particularly problematic because IgG4-RD can usually be treated effectively with medications such as corticosteroids, potentially avoiding the need for surgery if the correct diagnosis is made before the operation^[^9^]^.

We report the case of a 49-year-old man who underwent right radical orchiectomy for a suspected testicular tumor. Microscopic examination and special staining revealed a noncancerous lesion with a high proportion of IgG4-positive plasma cells (40–45%), confirming the diagnosis of IgG4-related disease. This case contributes to the small number of reported testicular IgG4-RD cases and highlights the importance of considering this diagnosis to prevent unnecessary surgical removal of the testicle.

This case was written in accordance with the 2025 SCARE guidelines^[^10^]^.

## Case presentation

A 49-year-old male from eastern Nepal presented to the urology outpatient department with a 3-day history of right testicular swelling. The swelling was painless, and there was no history of trauma, fever, burning micturition, weight loss, or systemic symptoms. His past surgical history was notable for a laparoscopic appendectomy performed 11 years ago. There was no known history of tuberculosis, autoimmune conditions, or malignancy. On physical examination, the patient was oriented, with stable vital signs. His general condition was fair. Abdominal examination was unremarkable: soft, non-distended, and non-tender with normal bowel sounds. Local scrotal examination revealed an enlarged, firm right testis, non-tender, with no skin changes or inguinal lymphadenopathy. The left testis was normal on palpation.

Laboratory tests showed a hemoglobin level of 15 g/dL, a total leukocyte count of 7900/cumm, and a platelet count of 277 000/cumm. Serum sodium and potassium were 140 and 3.8 mEq/L, respectively. Tumor markers were within normal limits: beta-human chorionic gonadotropin (β-hCG) 1.2 IU/L, alpha-fetoprotein (AFP) 1.19 ng/mL, lactate dehydrogenase (LDH) 178 IU/L, and prostate-specific antigen 0.78 ng/mL. Urine culture showed no growth.

Preoperative serum IgG4 was not measured. In retrospect, this represents a recognized limitation: given the clinical setting and the elevated suspicion of malignancy, this investigation was not considered preoperatively. This is consistent with the broader literature, which reports that serum IgG4 is frequently overlooked in patients presenting with isolated testicular masses, as the index of suspicion for IgG4-RD is low relative to that for germ cell tumors.

**Figure 1.**
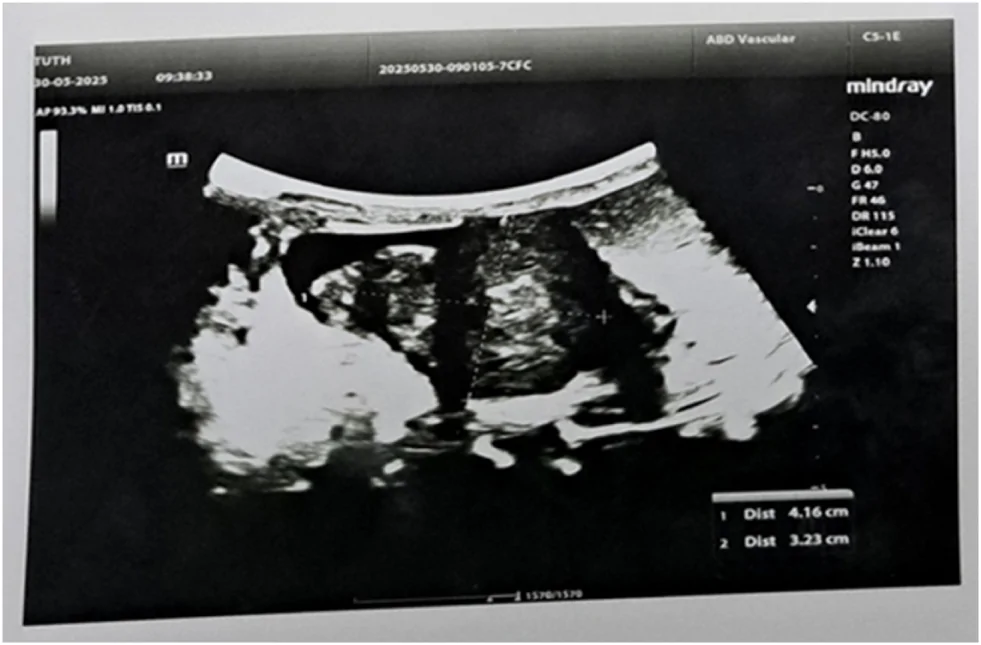
Scrotal ultrasonography revealed multiple hypo- to heteroechoic lesions in the right scrotal sac without internal vascularity.

Scrotal ultrasonography revealed multiple hypo- to heteroechoic lesions in the right scrotal sac without internal vascularity, with a bulky right epididymis and a simple cyst. The left testis showed a small simple cyst (Fig. 1).

Fine-needle aspiration cytology from the right testis showed cells in loose clusters, aggregates, and scattered singly, with round-to-oval nuclei, granular chromatin, moderately eosinophilic to vacuolated cytoplasm, stromal fragments, and scattered lymphocytes. Ziehl–Neelsen staining was negative for acid-fast bacilli.

The cytological impression was of a benign testicular tumor, with an adenomatoid tumor as the primary differential diagnosis. An excisional biopsy was recommended for confirmation. An ultrasound of the abdomen and pelvis showed bilateral simple renal cortical cysts and grade I prostatomegaly.

Based on the presentation of a painless, firm testicular mass with suspicious imaging findings and normal tumor markers, several differential diagnoses were considered. A testicular germ cell tumor, particularly seminoma, remained a possibility despite the normal levels of β-hCG, AFP, and LDH. Benign testicular tumors such as epidermoid cysts or adenomatoid tumors were also considered. Tuberculous orchitis was included in the differential, though there was no supporting systemic or laboratory evidence. A rarer consideration was testicular involvement by IgG4-related disease, given the atypical features and absence of constitutional symptoms. Sarcoidosis was a remote consideration in the differential, deemed less likely due to the absence of systemic manifestations. Despite the benign cytology and normal tumor markers, the irregular and nodular appearance of the testis on imaging raised a significant concern for an underlying malignancy, leading to the decision to proceed with orchiectomy.

**Figure 2.**
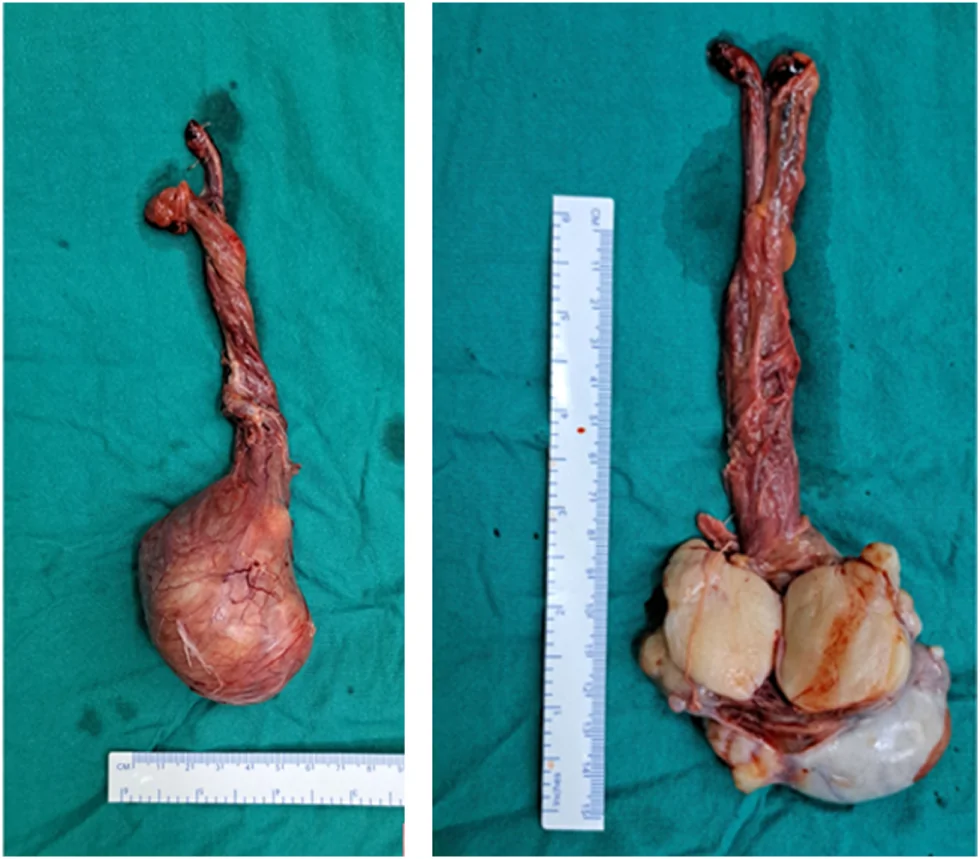
The right testis was irregular with multiple nodular growths and appeared small. No gross evidence of tumor extension was noted beyond the tunica.

The patient underwent a right high inguinal orchiectomy. Intraoperatively, the right testis was found to be irregular, with multiple nodular growths, and it appeared small in size. No gross evidence of tumor extension was noted beyond the tunica (Fig. 2). Histopathological examination revealed dense lymphoplasmacytic infiltration with storiform fibrosis, and an absence of malignancy. Immunohistochemistry showed a high proportion of IgG4-positive plasma cells (>40–45%), confirming the diagnosis of IgG4-related disease of the testis. The patient had an uneventful postoperative recovery.

The patient was discharged on the second postoperative day with oral antibiotics, analgesics, and advice for scrotal support. Following histopathological confirmation of IgG4-related disease, the serum IgG4 level measured 4 weeks after surgery was within normal limits. Further radiological evaluation for systemic involvement with contrast-enhanced CT was advised but could not be performed because of financial constraints. Clinical assessment during follow-up revealed no symptoms or signs suggestive of involvement of the salivary glands, lacrimal glands, pancreas, hepatobiliary system, lungs, kidneys, or retroperitoneum. In view of complete surgical excision, a normal postoperative serum IgG4 level, and the absence of clinical evidence of active disease, corticosteroid therapy was not initiated. At the 12-month follow-up, the patient remained asymptomatic with no clinical evidence of recurrence, contralateral testicular involvement, or symptoms suggestive of extra-testicular IgG4-related disease. He continues to undergo periodic urological surveillance.

## Discussion

IgG4-RD is a systemic fibroinflammatory process that may involve nearly any organ system. Although now being described with increasing frequency in the genitourinary system, isolated testicular involvement remains a very rare and diagnostically confusing presentation^[^11^]^. Our case of a 49-year-old man who underwent orchiectomy for a suspected tumor, which was ultimately diagnosed as IgG4-RD, highlights this pitfall and underscores the overriding importance of heightened clinical awareness.

Our patient’s presentation differs from the typical IgG4-RD presentation in several significant aspects. Firstly, the disease most frequently affects patients over the age of 50^[^12^]^, making our patient, at 49 years old, slightly younger than typical. Secondly, if IgG4-RD is a single-organ disease, the pancreas or salivary glands are the most likely to be involved; thus, primary gonadal involvement is unusual. Additionally, the painless, firm mass of acute onset in our case differs from the scrotal pain and more indolent, diffuse swelling commonly reported in most cases of testicular IgG4-RD^[^13^]^. Finally, the synchronous involvement of both the epididymis and the testis in our case is another feature of its rarity, since combined involvement is very rare^[^14^]^.

The greatest challenge in the treatment of testicular IgG4-RD is its high propensity to disguise itself as other conditions, which is frequently misdiagnosed as malignancy. As in our case, it most frequently presents as a tumor-like mass, invoking a high index of suspicion for germ cell tumors such as seminoma^[^15^]^. However, the absence of internal vascularity on Doppler ultrasonography and normal serum tumor markers (β-hCG, AFP, LDH) were crucial evidence against this diagnosis^[^16^]^. Beyond malignancy, the differential diagnosis is broad. Testicular tuberculosis was considered but deemed unlikely in the absence of chronic pain, systemic symptoms, or microbiological evidence^[^17^]^. Sarcoidosis can present similarly with a firm, painless mass, but was less likely without characteristic systemic manifestations^[^18^]^. Benign lesions, such as adenomatoid tumors and epidermoid cysts, were also part of the initial differential due to the cytological impression and clinical presentation^[^18^]^. It was exactly this diagnostic dilemma, in which clinical and radiological suspicion of malignancy was high despite inconclusive tumor marker findings, that led to the decision for surgical intervention.

A notable limitation in our case was the failure to obtain preoperative serum IgG4 levels. In a resource-limited referral setting with a dominant clinical impression of malignancy, this investigation was not pursued. Critically, normal serum IgG4 does not exclude the diagnosis: up to two-thirds of patients with single-organ IgG4-RD may have serum IgG4 within the reference range^[^19^]^, and the absence of serological confirmation is therefore not itself a barrier to diagnosis. Nevertheless, the oversight underscores the value of including IgG4-RD in the preoperative differential for any painless testicular mass with normal tumor markers, particularly when Doppler imaging shows absent internal vascularity.

The definitive diagnosis in our patient was established through histopathological examination and the use of the 2020 Revised Comprehensive Diagnostic criteria for IgG4-RD. According to these criteria, a definitive diagnosis is established if all three features are fulfilled: (1) clinical/radiological findings of organ enlargement, (2) elevated serum IgG4 level (>135 mg/dL), and (3) histopathological findings of dense lymphoplasmacytic infiltration, storiform fibrosis, and an IgG4 +/IgG + plasma cell ratio >40%^[^20^]^. Our case met criteria 1 and 3, constituting a “probable” diagnosis. While preoperative serum IgG4 levels were not measured, it is now well established that as many as two-thirds of patients with IgG4-RD, particularly those with single-organ disease, can have normal serum IgG4 levels^[^21^]^. Therefore, in clinical practice, a characteristic histopathological picture, with malignancy excluded, is accepted as sufficient for diagnosis, even in the absence of serological confirmation.

Testicular IgG4-RD management is a profound paradox. Glucocorticoids are the foundational first-line remission-inducing therapy, and steroid-sparing agents like rituximab or conventional disease-modifying antirheumatic drugs (e.g., azathioprine, mycophenolate mofetil) are used for remission maintenance and to reduce the high relapse rates that occur with steroid monotherapy^[^22^]^.

Although glucocorticoids remain the standard first-line treatment for active IgG4-RD, management should be individualized according to disease burden, organ involvement, symptoms, and evidence of ongoing inflammatory activity^[^21,22^]^. In our patient, corticosteroid therapy was not initiated because the lesion had been completely excised via orchiectomy, postoperative serum IgG4 levels were within the normal range, and there was no clinical evidence of residual or systemic disease during follow-up. This approach is supported by previous reports describing prolonged remission in selected patients with localized gonadal IgG4-RD managed without corticosteroid therapy^[^12^]^. At 12 months of follow-up, our patient remained asymptomatic without evidence of recurrence or extra-testicular involvement, suggesting that careful surveillance may be a reasonable strategy in selected cases following complete surgical excision.

Despite the availability of successful medical treatment, the reality is that most reported cases, including ours, are diagnosed postorchiectomy. A literature review by Wang *et al* that included 18 cases demonstrated that the majority of patients (11 of 18) were treated with surgery alone, illustrating how the masquerading nature of the disease leads to permanent surgical intervention^[^14^]^. This demonstrates a wide preoperative diagnostic gap.

## Conclusion

Isolated testicular IgG4-related disease is an exceptionally rare entity that can closely mimic testicular malignancy. Normal tumor markers and atypical imaging features should raise suspicion for a benign inflammatory process. Histopathological evaluation with IgG4 immunostaining is essential for diagnosis, allowing appropriate medical management with corticosteroids and potentially avoiding unnecessary orchiectomy. Early recognition and inclusion of IgG4-RD in the differential diagnosis are critical for improving patient outcomes and preserving testicular function.

## Data Availability

The data that support the findings of this study are available from the corresponding author upon reasonable request.

## Key clinical message

Isolated testicular IgG4-related disease is a rare but distinct mimic of testicular cancer. Clinicians should recognize that normal tumor markers and avascular hypoechoic lesions may indicate a benign inflammatory process rather than malignancy. Early histopathological and immunohistochemical evaluation for IgG4 positivity can establish the diagnosis and guide appropriate management, which may range from surveillance to immunosuppressive therapy depending on the clinical context.
